# Screening for Atrial Fibrillation Using Digital Health: Moving From Promises to Reality∗

**DOI:** 10.1016/j.jacadv.2023.100621

**Published:** 2023-09-18

**Authors:** Meghan Reading Turchioe, David Slotwiner

**Affiliations:** a Columbia University School of Nursing, New York, New York, USA; b Department of Cardiology, NewYork-Presbyterian Queens, Queens, New York, USA; c Department of Population Health Sciences, Weill Cornell Medicine, New York, New York, USA

**Keywords:** atrial fibrillation, digital technology, electrocardiography, screening

**Figure:**
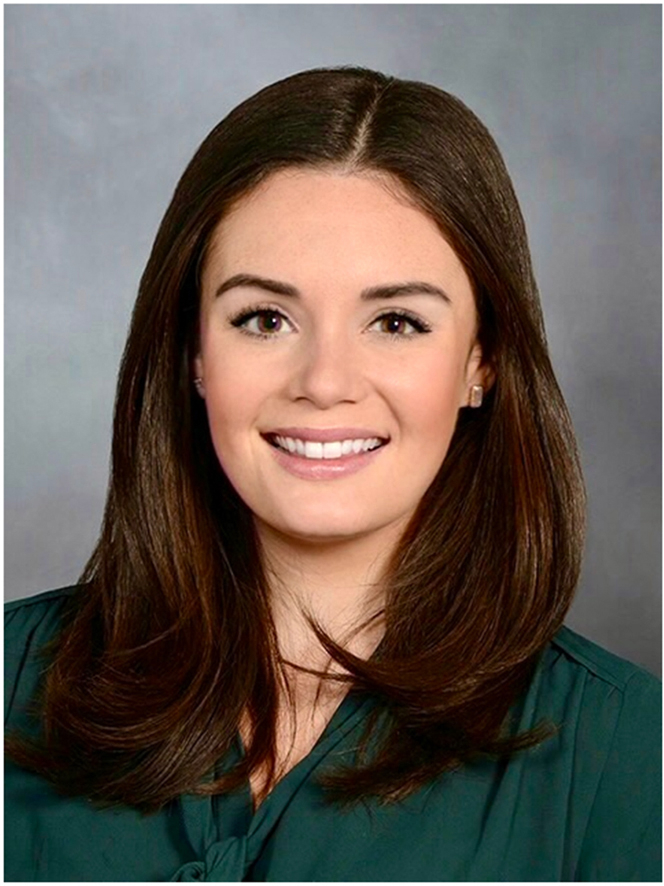

The past decade has brought a rapid increase in consumer-facing digital health tools that can detect and monitor atrial fibrillation (AF). These include wearable smartwatches, consumer-grade devices that pair with smartphones, and smartphone applications that leverage photoplethysmography technology. Digital health tools have generated excitement about the potential for broader AF screening because of their accessibility, affordability, and scalability compared to prior methods of detection. However, evidence demonstrating how these tools should be used in general populations without known AF, and what their diagnostic utility is, remains limited.

In this issue of *JACC: Advances*, Khurshid et al^1^ began to address this question when they evaluated the accuracy of electrocardiogram (ECG) tracings from AliveCor’s Kardia device from over 14,000 adults being screened for AF in primary care settings in the context of the VITAL-AF trial.^2^ Using trained medical assistants to obtain a recording and a cardiologist to overread as the gold standard, they reported that tracings classified as normal by AliveCor’s algorithm had a negative predictive value of 99.8%. However, tracings classified as possible AF only had a positive predictive value of 51.7%, and 20% of tracings could not be classified by AliveCor’s algorithm—4% to 8% of these equivocal tracings were later deemed AF by a cardiologist. These findings suggest that we should consider refocusing point-of-care digital health interventions on *screening out* rather than *screening in* AF; positive or equivocal results should be referred for further follow-up.

This study, therefore, generates practical evidence about how these tools may tangibly be used in a medical setting for AF surveillance. Primary care practices are frequently busy, with limited time and resources to be able to conduct routine 12-lead ECGs on all older adults. Digital health devices such as Kardia are more feasible and cheaper (Kardia, eg, costs under $100 per device) to implement at scale. The VITAL-AF trial used a version of Kardia that records a single-lead ECG, but the U.S. Food and Drug Administration recently cleared the technology for 6-lead ECG recording as well.^3^ This makes Kardia and other Food and Drug Administration-cleared digital health tools^4^ appealing options for clinicians to quickly and easily identify potentially at-risk groups (eg, those with equivocal or possible AF tracings) who may benefit from more intensive monitoring.

Scalable, accurate digital health tools for population surveillance of AF are of dire importance. One study estimated that 13% of true AF cases go undiagnosed and more than one-half (56%) of these cases have an elevated stroke risk warranting anticoagulation (CHADS_2_ score ≥2).^5^ This helps to explain the well-known statistic that 20% of new AF diagnoses occur in patients presenting with ischemic strokes.^6^ Therefore, improved AF screening has the potential to increase the percentage of patients who receive appropriate thromboembolic prophylaxis and thus reduce the incidence of strokes among the many high-risk yet undiagnosed cases. Unfortunately, clinical trials of digital health-based AF screening often produce low yields of newly diagnosed AF, even when risk-stratified screening methods are used^7^, including in the parent VITAL-AF trial. In VITAL-AF, the investigators conducted a randomized, controlled trial comparing the effect of point-of-care Kardia-based screening in primary care practices to usual care on new diagnoses of AF among older adults. They determined that there were no differences in new diagnoses of AF or anticoagulation initiation between the groups.^2^

Screening for AF in older adults remains an important priority. Older adults are at higher risk for AF, with prevalence increasing approximately 5% per year after age 65 years.^8^ Clinical consensus guidelines provide guidance on AF screening in adults age 65 and older but do not address how digital health should be used in this context.^9^^,^^10^ In VITAL-AF, Khurshid et al^1^ used trained medical assistants to obtain each ECG recording. This is not representative of how consumer digital health tools are typically used and may have resulted in higher-quality recordings than would be observed in the general population. The 2022 U.S. Preventive Services Task Force Recommendations for Screening for Atrial Fibrillation point out that digital health technologies have led to even greater detection of intermittent AF of short duration, which has unclear treatment benefits given the potential risks of oral anticoagulation in older adults.^11^^,^^12^ These important questions were not addressed in VITAL-AF or the follow-up study from Khurshid et al.^1^ Importantly, Khurshid et al^1^ reported that the proportion of equivocal results increased with age, and approximately one-quarter of tracings were equivocal for adults aged 80 and older. The investigators attribute these results to the higher proportion of physical challenges (such as tremors and decreased dexterity) and other non-AF atrial arrhythmias in older adults that are known to decrease the Kardia algorithm’s accuracy. To make meaningful strides towards understanding the clinical utility of digital health in older adults, both improved algorithm accuracy in these populations and rigorous trials demonstrating appropriate treatment based on subclinical, digital health-detected AF are needed.

Finally, we agree with Khurshid et al^1^ that more work is needed to understand the potential to use digital health for AF screening among individuals who are less likely to receive routine AF prevention and care, such as racial and ethnic minority individuals. A limitation of this study is its lack of racial diversity—just 5% of participants were Black—thereby limiting the conclusions that can be drawn from these individuals. In fact, most digital health studies of AF have also included predominantly White samples; of the Apple Heart study’s 419,000 participants, only 8% were Black.^13^ Black adults may be less likely to receive a diagnosis of AF even when a similar AF burden is documented on ambulatory ECG monitors compared to White participants, as was observed in the MESA (Multi-Ethnic Study of Atherosclerosis),^14^ although this has remained an open question due to mixed findings in other studies.^15^ Nonetheless, it is well known that Black patients are less likely to receive guideline-directed AF treatment and more likely to experience AF-related strokes compared to White individuals,^15^ demonstrating the opportunities to improve the detection and management of AF in these populations. A greater emphasis on racial diversity in future clinical trials leveraging digital health for AF screening is critically needed.

## Funding support and author disclosures

Dr Turchioe is supported by the 10.13039/100000056National Institute of Nursing Research (NINR/NIH) under award number R00NR019124; has done consulting for Boston Scientific; and has equity in and Iris OB Health. All other authors have reported that they have no relationships relevant to the contents of this paper to disclose.

## References

[1] Khurshid S., Chang Y., Borowsky L.H. (2023). Performance of single-lead handheld electrocardiograms for atrial fibrillation screening in primary care: the VITAL-AF trial. JACC: Adv.

[2] Lubitz S.A., Atlas S.J., Ashburner J.M. (2022). Screening for atrial fibrillation in older adults at primary care visits: VITAL-AF randomized controlled trial. Circulation.

[3] (2019). FDA grants first ever clearance for six-lead personal ECG device. Alivecor. https://www.alivecor.com/press/press_release/fda-grants-first-ever-clearance-for-six-lead-personal-ecg-device.

[4] Ding E.Y., Marcus G.M., McManus D.D. (2020). Emerging technologies for identifying atrial fibrillation. Circ Res.

[5] Turakhia M.P., Shafrin J., Bognar K. (2018). Estimated prevalence of undiagnosed atrial fibrillation in the United States. PLoS One.

[6] Jaakkola J., Mustonen P., Kiviniemi T. (2016). Stroke as the first manifestation of atrial fibrillation. PLoS One.

[7] Khurshid S., Healey J.S., McIntyre W.F., Lubitz S.A. (2020). Population-based screening for atrial fibrillation. Circ Res.

[8] Piccini J.P., Hammill B.G., Sinner M.F. (2012). Incidence and prevalence of atrial fibrillation and associated mortality among Medicare beneficiaries, 1993-2007. Circ Cardiovasc Qual Outcomes.

[9] January C.T., Wann L.S., Calkins H. (2019). 2019 AHA/ACC/HRS focused update of the 2014 AHA/ACC/HRS guideline for the management of patients with atrial fibrillation: a report of the American College of Cardiology/American Heart Association Task Force on Clinical Practice Guidelines and the Heart Rhythm Society in Collaboration with the Society of Thoracic Surgeons. Circulation.

[10] Kirchhof P., Benussi S., Kotecha D. (2016). 2016 ESC Guidelines for the management of atrial fibrillation developed in collaboration with EACTS. Eur Heart J.

[11] Davidson K.W., Barry M.J., US Preventive Services Task Force (2022). Screening for atrial fibrillation: US Preventive Services Task Force recommendation statement. JAMA.

[12] Greenland P. (2022). Screening for atrial fibrillation—more data still needed. JAMA.

[13] Perez M.V., Mahaffey K.W., Hedlin H. (2019). Large-scale assessment of a smartwatch to identify atrial fibrillation. N Engl J Med.

[14] Heckbert S.R., Austin T.R., Jensen P.N. (2020). Differences by race/ethnicity in the prevalence of clinically detected and monitor-detected atrial fibrillation. Circ Arrhythm Electrophysiol.

[15] Tamirisa K.P., Al-Khatib S.M., Mohanty S. (2021). Racial and ethnic differences in the management of atrial fibrillation. CJC Open.

